# Late Solitary Pancreatic Metastasis from Renal Cell Carcinoma: A Case Report

**DOI:** 10.1155/2012/464808

**Published:** 2012-06-26

**Authors:** Anastasios Katsourakis, George Noussios, Iosif Hadjis, Michael Alatsakis, Efthimios Chatzitheoklitos

**Affiliations:** ^1^Department of Surgery, Agios Dimitrios General Hospital, 54634 Thessaloniki, Greece; ^2^Laboratory of Anatomy, Department of Physical Education and Sports Sciences at Serres, Aristotelian University, 54124 Thessaloniki, Greece

## Abstract

We report a case of a 70-year-old man with renal cell carcinoma and metastasis to the pancreas. Symptomatic patients usually present with obstructive jaundice, abdominal pain, or GI bleeding. The diagnosis usually occurs in asymptomatic patients during followup for renal cell carcinoma. It usually befalls slowly from 2 to 18 years after the onset of the primary tumor of the kidney. A 70-year-old man presented in our department with weight loss, anorexia, and elevated blood glucose, having a large tumor on the head of the pancreas treated successfully by pancreatoduodenectomy. Three years after his treatment, the patient is doing well and without recurrence of the tumor. In conclusion, metastasis of renal cell carcinoma to the pancreas is a rare neoplasm accounting for 0.25–3% of all pancreatic tumors.

## 1. Introduction

 Metastatic cancer of the pancreas from another primary site is rare. Renal cell cancer, along with malignant melanoma, lung, colon and breast carcinoma, is among the few tumors known to metastasize to the pancreas [1]. It has a late onset of 2 to 18 years after the occurrence of the primary tumor. Usually it coexists with metastases of the lungs, brain, or bones. When it is solitary to the pancreas, it is distributed equally to all parts of it (head, body, and tail) [2]. The metastasis of the kidney occurs via blood or lymph vessels, although no infiltration of the peripancreatic lymph nodes exists. Diagnostic imaging fails to differentiate between primary pancreatic tumors. On account of a very late metastatic onset, we present a case of a 70-year-old patient with a solitary metastatic tumor of the pancreas 22 years after right nephrectomy due to renal cell carcinoma.

## 2. Case Report

**Figure 1:**
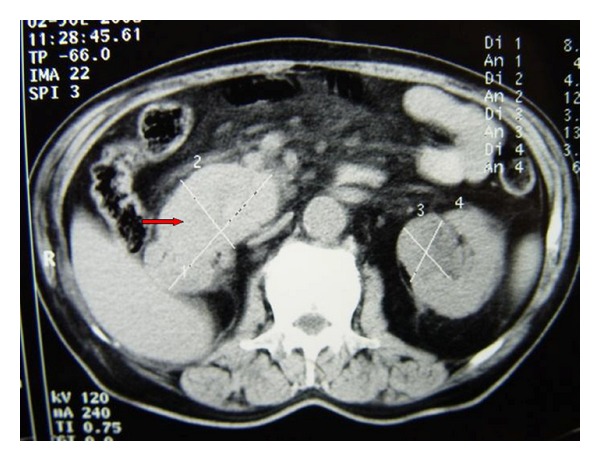
CT examination of the abdomen revealed a mass at the pancreatic head (arrow).

A 70-year-old male presented to our outpatient department with anorexia and weight loss during the last three months. He was diagnosed with myelodysplastic syndrome a year ago and was treated accordingly by a hematologist. The physical examination was noncontributory. The medical history of the patient revealed right nephrectomy 22 years ago owing to renal cell carcinoma; grade 2 (according to Fuhrmann grading system) and T1b (TNM staging system for kidney cancer, 7th edition). His blood glucose was elevated. Liver function tests, serum amylase, bilirubin; CEA and CA 19-9 were within the normal limits. Ultrasound examination of the abdomen revealed a large mass at the region of the pancreatic head. Contrast-enhanced computed tomography of the abdomen was performed, which confirmed a 9 × 5 cm lesion at the head of the pancreas (Figure 1). Due to his medical history of a right nephrectomy 22 years ago owing to renal cell carcinoma, a CT scan of the brain and the thorax was performed, which was negative for metastases. 

**Figure 2:**
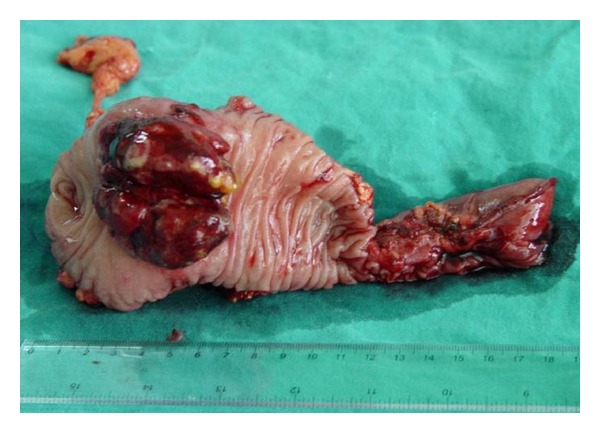
Gross appearance of the tumor.

**Figure 3:**
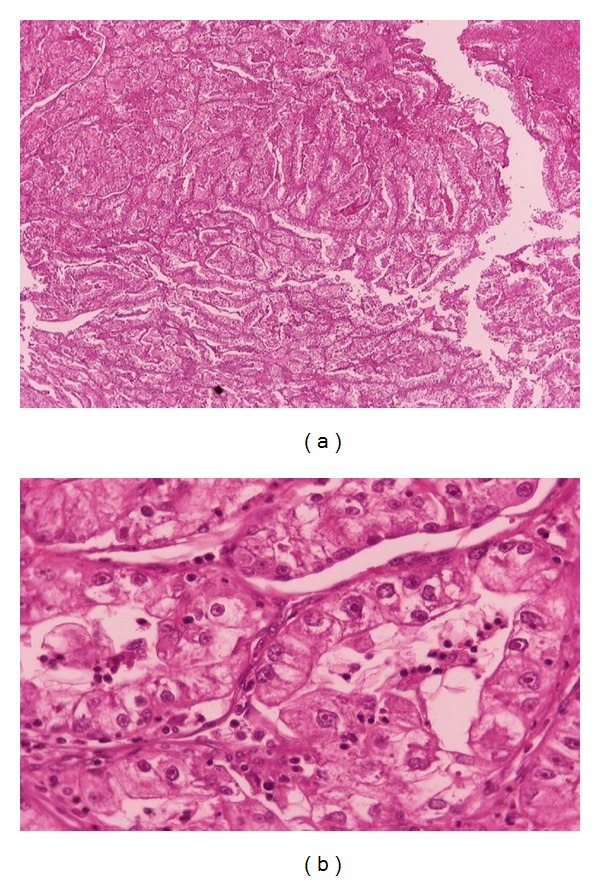
(a) Histology of the resected pancreatic specimen showing clear cells of renal cell carcinoma, capsule and normal pancreas. (b) Histology of the primary resected renal cell carcinoma showing the characteristic findings of large clear cells.

The patient underwent a pancreatoduodenectomy with pylorus preserving (Longmire-Traverso) in order to remove the tumor. The postoperative period was uneventful, and the patient was released from our department 7 days after his operation. Histopathological evaluation showed a 9 × 5 × 4 cm solid lesion with areas of hemorrhage and necrosis (Figure 2). The lesion was composed of solid sheets of cells divided by bands of fibrovascular tissue into large nests and alveoli. The cells showed moderate clear-to-granular eosinophilic cytoplasm with well-defined cell borders. The nuclei were central and pleomorphic with conspicuous nucleoli. The lesion was surrounded by a thick collagenous capsule and completely separated from the pancreatic tissue with no infiltration. The morphology was identical to that of the primary renal tumor which was reviewed. A final diagnosis of metastatic clear cell renal cell carcinoma of the head of the pancreas was reached (Figures 3(a) and 3(b)). The surgical margins were free of tumor. The followup of the patient was every 6 months for the first 2 years and then annually. The patient remains free of symptoms and without recurrence of the primary tumor three years after his treatment.

## 3. Discussion

 Metastasis of the pancreas is very rare. Only 4.5% of all pancreatic tumors are metastatic. Pancreatic metastases from renal cell carcinoma are rare and usually present themselves many years after primary diagnosis [1, 3]. They are accompanied by metastatic lesions in the brain, lungs, or bones. Solitary tumors in the pancreas are extremely rare (0.3% of all pancreatic tumors). They are very difficult to be diagnosed because existing imaging cannot differentiate them from adenocarcinoma of the pancreas [4]. Thus, the patient's history is very important. These patients could be asymptomatic. In a review of 236 cases in the literature, Sellner et al. [5] reported 35% of these patients to be asymptomatic with others presenting with symptoms which included abdominal pain (20%), GI bleeding due to duodenal infiltration (20%), obstructive jaundice (9%), weight loss (9%), and pancreatitis and diabetes (3% each). 

The mode of spread of renal cell carcinoma to the pancreas is controversial and can either be hematogenous or via lymphatics with direct spread to the pancreas being unusual. Spread through lymphatics may occur by retrograde lymph flow secondary to tumor infiltration of the retroperitoneal lymph nodes. Hematogenous spread may occur along the draining collateral vein of a hypervascular renal tumor with or without associated renal vein thrombosis [6].

 Surgical resection of primary renal cell carcinoma and metastatic deposits remain the most effective treatment since chemotherapy, radiotherapy, and hormonal therapy have generally proved ineffective. Resection of a pancreatic metastasis may involve a standard pancreaticoduodenectomy or a distal pancreatectomy depending on the location of the secondary deposit. Atypical resection of pancreatic metastasis from renal cell carcinoma, such as duodenum-preserving pancreatic head resection, middle pancreatectomy, and enucleation of the tumor, has been adopted by some authors. Atypical resection has been adopted based on the fact that these lesions are well encapsulated [1, 6–8]. Five-year survival ranges from 29% to 81%. Multiple metastatic lesions have worse prognosis than solitary ones. The best treatment is R0 resection of the tumor which is curative in most cases of solitary tumors [9, 10]. 

 In conclusion, solitary pancreatic metastasis from renal cell carcinoma is a rare pancreatic tumor. It is usually presented years after the primary diagnosis and it is very difficult to differentiate it from pancreatic adenocarcinoma. The only possible curative treatment is R0 resection.

## References

[1] Thompson LD, Heffess CS (2000). Renal cell carcinoma to the pancreas in surgical pathology material: a clinicopathologic study of 21 cases with a review of the literature. *Cancer*.

[2] Sohn TA, Yeo CJ, Cameron JL, Nakeeb A, Lillemoe KD (2001). Renal cell carcinoma metastatic to the pancreas: results of surgical management. *Journal of Gastrointestinal Surgery*.

[3] Crippa S, Angelini C, Mussi C (2006). Surgical treatment of metastatic tumors to the pancreas: a single center experience and review of the literature. *World Journal of Surgery*.

[4] Robbins EG, Franceschi D, Barkin JS (1996). Solitary metastatic tumors to the pancreas: a case report and review of the literature. *American Journal of Gastroenterology*.

[5] Sellner F, Tykalsky N, de Santis M, Pont J, Klimpfinger M (2006). Solitary and multiple isolated metastases of clear cell renal carcinoma to the pancreas: an indication for pancreatic surgery. *Annals of Surgical Oncology*.

[6] David AW, Samuel R, Eapen A, Vyas F, Joseph P, Sitaram V (2006). Pancreatic metastasis from renal cell carcinoma 16 years after nephrectomy: a case report and review of the literature. *Tropical Gastroenterology*.

[7] Zerbi A, Ortolano E, Balzano G, Borri A, Beneduce AA, di Carlo V (2008). Pancreatic metastasis from renal cell carcinoma: which patients benefit from surgical resection?. *Annals of Surgical Oncology*.

[8] Wente MN, Kleeff J, Esposito I (2005). Renal cancer cell metastasis into the pancreas: a single-center experience and overview of the literature. *Pancreas*.

[9] Tuech JJ, Pessaux P, Chautard D (1999). Results of duodenopancreatectomy for solitary pancreatic metastasis from renal cell carcinoma. *Journal of Hepato-Biliary-Pancreatic Surgery*.

[10] Kavolius JP, Mastorakos DP, Pavlovich C, Russo P, Burt ME, Brady MS (1998). Resection of metastatic renal cell carcinoma. *Journal of Clinical Oncology*.

